# LINC00152 Promotes Tumor Progression and Predicts Poor Prognosis by Stabilizing BCL6 From Degradation in the Epithelial Ovarian Cancer

**DOI:** 10.3389/fonc.2020.555132

**Published:** 2020-11-12

**Authors:** Shunni Wang, Weiwei Weng, Tingting Chen, Midie Xu, Ping Wei, Jing Li, Linghui Lu, Yiqin Wang

**Affiliations:** ^1^ Department of Pathology, Obstetrics and Gynecology Hospital of Fudan University, Shanghai, China; ^2^ Department of Pathology, Fudan University Shanghai Cancer Center, Shanghai, China

**Keywords:** long non-coding RNA 00152, BCL6, ubiquitination, ovarian cancer, lncRNA

## Abstract

Long non-coding RNA 00152 (LINC00152) is tumorigenic in multiple somatic malignancies. However, its prognostic significance and molecular mechanisms in the epithelial ovarian cancer (EOC) remain elusive. Here our study reveals that dysregulation of LINC00152 is a predictor of poor prognosis in patients with EOC and facilitates ovarian tumor growth and metastasis both *in vitro* and *in vivo*; the expression of LINC00152 positively correlates with the protein levels of BCL6 in EOC tissues and ovarian tumor cells; LINC00152 binds to Ser333 and Ser343 of BCL6 protein and stabilizes BCL6 from poly-ubiquitination thus facilitating the oncogenic functions in EOC. Moreover, overexpression of the mutant BCL6^S333A/S343A^ fails to rescue the reduced proliferation and invasion caused by the knockdown of endogenous BCL6 in LINC00152-overexpressing cells. Our study might not only offer clues to the network of lncRNA-protein interactions but also provide potential therapeutic targets for the tumor pharmacology.

## Highlights

This work reveals that dysregulation of LINC00152 in ovarian cancer predicts poor survivals of patients, and the stabilization of BCL6 by LINC00152 promotes ovarian tumor proliferation and invasion.

## Introduction

Epithelial ovarian cancer (EOC) accounts for less than 3% of all malignancies in females, but is responsible for approximately 5% of total cancer deaths (1). Despite declining incidence and mortality in the past decades, inconspicuous symptoms and signs still lead to delayed diagnoses at advanced stages with pelvic dissemination, causing relatively low 5-year survival rates of 42% for stage III disease, and 26% for stage IV disease in the United States, respectively (2); this reveals a highly aggressive nature that needs elucidation of underlying molecular processes. So far, great efforts have been made to unveil the molecular aberrations in EOC, but only a few candidates have proven to be clinically applicable (3). Therefore, novel molecular targets for pharmacological selections are in demand.

The importance of the long non-coding RNAs (lncRNAs, non-coding RNAs with sizes larger than 200bps) are now widely recognized in cell differentiation (4), signal transduction (5) and somatic tumorigenesis and metastasis (6). The non-coding RNA 00152 (LINC00152) is a lncRNA located at 2p11.2 (7) and has been involved in cell proliferation (8), cell-cycle arrest (7), epithelial-mesenchymal transition (9) and cell invasion (10) in order to facilitate tumor initiation and progression. Our former studies and studies from other researches have revealed that LINC00152 promotes tumor invasion and predicts poor prognosis in renal clear cell (11), lung (12), gastric (13) and hepatic carcinoma (5); however, its role in the progression of EOC needs further exploration. The majority of lncRNAs serve as competing endogenous RNAs (ceRNAs) to fulfil their biological functions (14), as does LINC00152, which has been reported to regulated siRNAs in gastric and gallbladder cancers (15). Recently, lncRNAs have been reported to play a key role in the ubiquitination of proteins as well (16, 17). Ubiquitination is a posttranslational modification that requires the conjugation of ubiquitin (Ub) to the lysine residues of specific substrate proteins *via* the enzyme-mediated transfer (18), and is a crucial part of the molecular dysregulation in EOC (19). Since lncRNAs could either repress (20) or prompt (21) the ubiquitination of target substrates, it is unclear whether the molecular mechanisms of LINC00152 in EOC are linked to the ubiquitination of downstream proteins.

B-cell CLL/lymphoma 6 (BCL6) is a highly conserved zinc finger transcriptional factor and is implicated in the pathogenesis of human B-cell lymphomas by controlling the transcription of oncogenes (22). Previous studies demonstrate that BCL6 is degraded *via* the ubiquitin-proteosome system (23, 24) and its degradation is balanced by FBXO11 (23) and AIP (25) thus regulating the tumor growth of diffuse large B cell lymphoma. Recently BCL6 has also been reported to be dysregulated in somatic malignancies such as breast carcinoma (26, 27) and EOC (22, 28). However, it remains unclear whether degradation of BCL6 is related to lncRNAs and how the interaction is implicated in the tumorigenesis of somatic malignancies.

Here we found that LINC00152 is increased in EOC tissues and predicts poor clinical outcomes; LINC00152 mRNA level positively correlates with BCL6 protein in EOC tissues; LINC00152 binds to the Ser333/Ser343 of BCL6 and stabilizes its protein from ubiquitination. Furthermore, LINC00152-BCL6 interaction reinforces the oncogenic functions of BCL6 in ovarian tumor cells. Therefore, we believe that the modification on BCL6 by LINC00152 could be a potential therapeutic target for ovarian tumor pharmacology.

## Materials and Methods

### Patient Samples

152 pairs of tumor and paratumorous frozen tissues and paraffinized blocks with EOC were applied for this study from the biobank of Gynecological and Obstetrical Hospital of Fudan University from January 1^st^, 2012 to December 31^st^, 2016. The experiments were undertaken with the understanding and written consent of each subject and conformed to the standards set by the Declaration of Helsinki. None of the patients had received preoperative chemotherapy. The collected pathological features included age, tumor size, staging, opposite ovary and fallopian tube involvement, lymph node and remote metastasis, and recurrence. All patients were staged based on the International Federation of Gynecology and Obstetrics (FIGO) staging system 2014. The follow-up interval was from the date of surgery to the date of death or the last clinical investigation. This study was approved by The Clinical Research Ethics Committee of Gynecology and Obstetrics Hospital of Fudan University. Written informed consent was obtained from all participants.

### RNAScope Detection of LINC00152 mRNA and Immunofluorescence

Detection of LINC00152 mRNA was performed using the RNAscope 2.5 HD Detection Reagent-BROWN (#322310, Advanced Cell Diagnostics, USA) and Multiplex Fluorescent (#323100) according to the manufacturer’s instructions. The DapB probe (#310043) was used as the negative control and Hs-PPIB (#313901) as positive control. Probe-LINC00152 (#18081B) was used for the test samples.

Transfected A2780, SKOV3, and negative control cells were fixed with 4% paraformaldehyde for 1 h, penetrated with 0.5% Triton-100X (Sigma), and incubated with polyclonal rabbit anti-BCL6(Cellsignaling #14895) (1:100) and Probe-LINC00152 using the RNAscope RNA-protein confocal staining system. Then the cells were stained with Alexa Fluor 546 (red) along with Alexa Fluor 488 (green) (ACD, U.S.A.) as well as DAPI (Invitrogen, U.S.A.) for DNA staining.

Images were taken at 40x and 80x magnification using the BX45 (Olympus, Japan) light microscope, Leica inverted fluorescence microscope with ProgRes Image Capture Software (JENOPTIK Optical System, Jena, German) & Leica Confocal LAS-AF SP5 System. Dark-brown, punctuate dots in the nucleus and/or cytoplasm under the light microscope, and green (Fluor 488) signals under the fluorescence systems were considered positive for LINC00152. Red (Fluor 546) signals were considered positive for BCL6. The slides were evaluated by two pathologists blinded to the morphological diagnoses in order to exclude any possible influences of the morphology.

### *In Vivo* Xenograft and Metastasis Models

Animal experiments were approved by the Shanghai Medical Experimental Animal Care Commission and carried out in accordance with Fudan university laboratory animals administration. Female BALB/c-nu mice (4–5 weeks of age, 18-20g) were maintained under specific pathogen-free conditions in the Experimental Animal Department of Fudan University. All of the experimental procedures involving animals were undertaken in accordance with the institute guidelines. 1 × 10^7^ Lenti-NC, Lenti-LINC00152, Lenti-shC, Lenti–shLINC00152, Lenti-LINC00152-NC(BCL6), Lenti-BCL6-NC (LINC00152 Lenti-LINC00152-BCL6, and Lenti-LINC00152-BCL6^S333A/S343A^ stably infected SKOV3 cells were injected s.c. into the flank regions of 8-week old BALB/c female nude mice (n = 4 per group) and allowed to grow for 24–30 days. All the mice were euthanized and the xenografts were excised out and measured. The tumor volumes were calculated using the formula 1/2 × r1^2^ × r2 (r1 < r2).

For the intraperitoneal tumor metastasis models, 8 × 10^6^ SKOV3 cells stably infected with Lenti-NC or Lenti-LINC00152 were injected intraperitoneally (i.p.) (n = 3 per group). The abdominal circumferences of the mice were measured every 2 days. All the mice were anesthetized and sacrificed. The xenografts were excised and measured. The tumor volumes were calculated using the formula 1/2 × *r*12 × *r*2 (*r*1 < *r*2). The intraperitoneal organs (livers, peritoneal membranes, and intestines, etc.) were excised and examined for the implanted lesions followed by sampling. All the tissues were fixed and embedded with paraffin. Hematoxylin& Eosin staining was used to observe the lesions through a microscope.

### Biotin Pull-Down Assays and Mass Spectrometry

The LINC00152 and its antisense plasmid were linearly cut, transcribed, and biotin-labeled *in vitro* with Bio-16-UTP (Life Technologies) using a MAXIscript T7 Transcription Kit (Life Technologies). Protein–RNA interactions were carried out using a Pierce Magnetic RNA-Protein Pull-Down Kit (Life Technologies) with the lysates of H293T and CAOV3 cells. The FLAG-BCL6 protein complexes were fractionated by SDS-PAGE, followed by Prussian blue staining. The Prussian blue-stained gel bands were treated with sequencing-grade trypsin (Lot. V5280, Promega, USA). The resulting peptides were analyzed by nano-HPLC-MS/MS with online desalting with a FAMOS autosampler. Electrospray ion trap MS was performed using an LTQ linear ion trap mass spectrometer (Thermo Finnigan). The fragment spectra were analyzed using the National Center for Biotechnology Information nonredundant protein database using Mascot (Matrix Science) and Sequest (Thermo Scientific). The base peak and the candidate proteins pulled down by LINC00152 were listed in the **Supplementary Information**.

### Wound-Healing and Transwell Assays

For the wound healing assay, cells were seeded with 10% fetal bovine serum culture until 80% confluent in 6-well plates. Then A2780 and SKOV3 cells were transfected with pcDNA3.1 or pcDNA3.1-LINC00152 while CAOV3 and ES-2 cells with either siControl or siLINC00152 siRNAs for 24 h. Then Cells were scraped off using a pipette tip and washed softly with PBS twice to remove the floating cells and debris. After washed by PBS, the cells were incubated with serum-free culture for 24 h to minimize the interference of cell proliferation. Representative images of cells migrating into the wounds were captured at 0 and 24 h in the same wounded region.

The Transwell assay was used to assess cell invasion (Corning Co. Ltd., USA). The lower chambers were pre-coated with 100 μL Matrix gel (#354234, BD Bioscience, USA) for 30 min. 24 h after transfection, cells were seeded on the upper chamber at 3.0 × 10^4^/well in serum-free medium to minimize the interference of cell proliferation. Medium containing 20% fetal bovine serum medium was applied to the lower chamber as chemo-attractant. After 24 h incubation at 37° C, cells which invaded through the matrix gel and adhered to the lower surface of the filter were fixed with ethanol, stained with 0.5% crystal violet, photographed at 200×, and counted at 400× in 10 different fields to determine the average number of cells (BX51, Olympus, Japan).

### Statistical Analysis

Each experiment was performed in triplicate, and survival data were shown as the median following time, and other data were presented as the mean ± SD. All statistical analyses were performed using SPSS 20.0 (IBM, SPSS, Chicago, IL, USA). Student’s t-test and one-way ANOVA were used in either two or multiple groups for statistical significance. Poison correlation and Spearman rank order were used to analyze the correlations; disease-free survival (DFS) and disease-specific survival (DSS) curves were calculated with the Kaplan-Meier method and were analyzed with the log-rank test. The DFS rate was calculated from the date of surgery to the date of progression (local and/or distal tumor recurrence) or the date of death. The DSS rate was defined as the length of time between the diagnosis and death or last follow-up. Univariate analysis and multivariate models were fit using a Cox proportional hazards regression model. All tests were two-sided, and *P* < 0.05 was considered statistically significant.

Other methods used in this study are listed the **Supplementary Information**.

## Results

### LINC00152 Is Upregulated in Ovarian Cancer and Predicts Poor Clinical Outcomes

**Figure 1:**
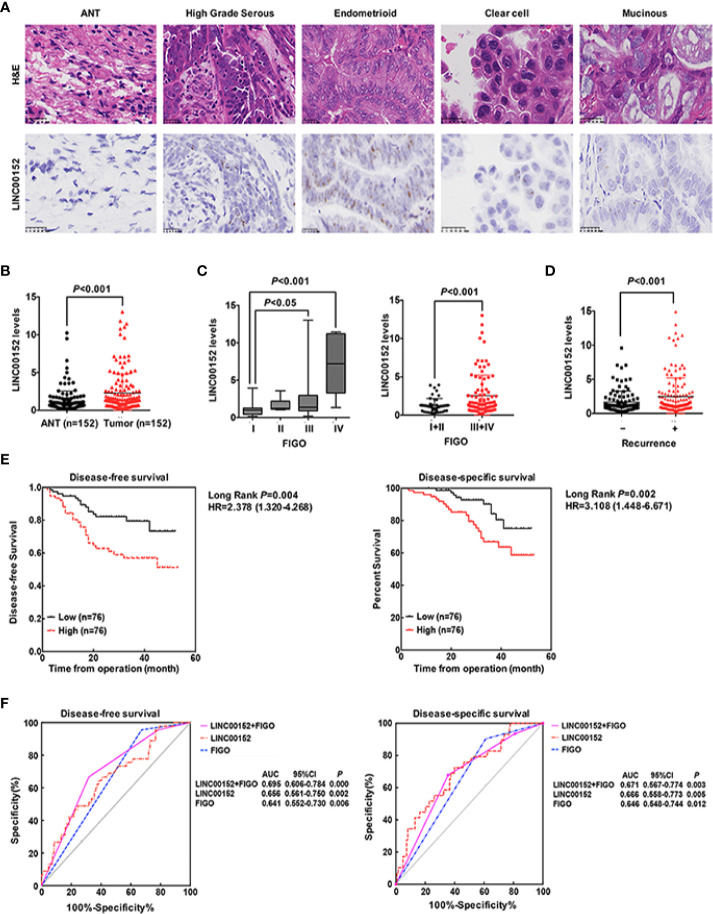
LINC00152 is upregulated in ovarian cancer and predicts poor clinical outcomes. **(A)** The expression of LINC00152 detected by *in situ* RNA hybridization in adjacent tumor tissues (ANTs) and different subtypes (high grade serous, endometrioid, clear cell and mucinous) of ovarian carcinomas. Scale bars = 25 µm. **(B)** The expression of LINC00152 detected by Real-time qPCR in 152 pairs of the adjacent tumor tissues (ANTs) and ovarian tumor tissues. *P* < 0.01. **(C)** The expression of LINC00152 detected by Real-time qPCR divided by FIGO stage in 152 cases of ovarian cancers. Left: The cases were divided into Stage I, II, III and IV. Right: The cases were divided into Stage I+II and Stage III+IV. *P* < 0.05. **(D)** The expression of LINC00152 detected by Real-time qPCR in cases with or without recurrences. *P* < 0.01. **(E)** The disease-free survivals (DFS) and the disease-specific survivals (DSS) of patients divided by expressions of LINC00152. The expression of LINC00152 was divided by the median value. The HR and the 95% CI were shown. *P* < 0.01. **(F)** The ROC curves for of LINC00152, FIGO and the combination of LINC00152 and FIGO for disease-free survivals (DFS) and the disease-specific survivals (DSS). The areas under curves (AUC) were shown. *P* < 0.01.

**Figure 2:**
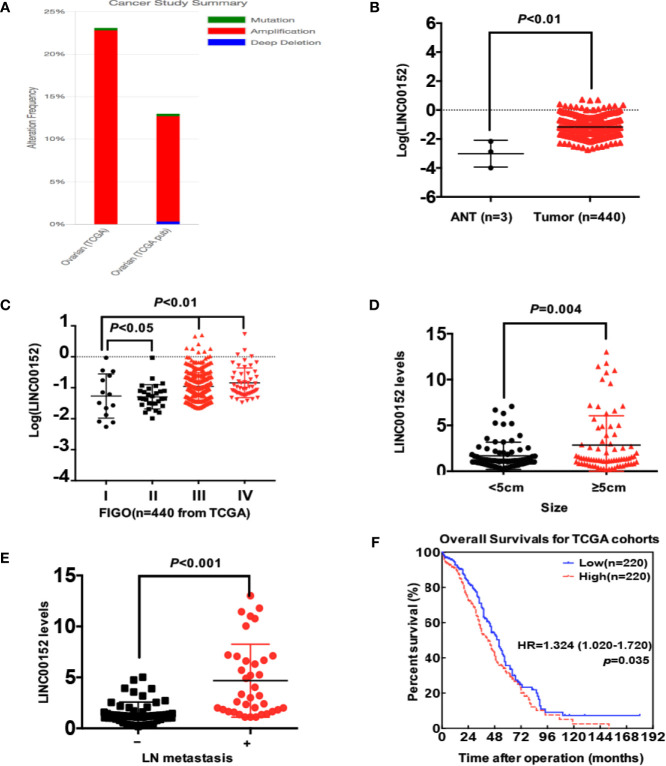
The statistical analysis of LINC00152 expression in TCGA data (www.cbioportal.org). **(A)** The summary of LINC00152 status in 2 independent cohorts from TCGA analyzed by www.cbioportal.org. The gene status included mutation, amplification and deep deletion. **(B)** The expression of LINC00152 in ovarian cancer tissues and adjacent normal tissues (ANTs) in cohorts from TCGA (n = 440). *P* < 0.01. **(C)** The expression of LINC00152 in the cases at different FIGO stages (n = 440) from TCGA. *P* < 0.05. **(D)** The comparison of LINC00152 expression in 152 cases with ovarian cancer divided by tumor sizes (<5 cm and ≥5 cm). *P* < 0.01. **(E)** The comparison of LINC00152 expression in 152 cases with ovarian cancer divided by the metastatic status of lymph nodes. *P* < 0.01. **(F)** The overall survivals (OS) of cases divided with the expression of LINC00152 in the cohorts from TCGA. *P* < 0.05.

We first detected the levels of LINC00152 mRNA using RNAscope and RT-qPCR in 152 pairs of EOC and adjacent normal tissues (ANTs). The RNAscope showed that LINC00152 was positively stained in the tumor lesions but was negative in the ANTs (**Figure 1A**). 64.65% of high-grade serous, 72.00% of endometrioid, 23.81% of clear cell and 28.57% of mucinous subtype showed positive expression of LINC00152 (**Figure 1A** and **Table 1**). The RT-qPCR results showed that the mRNA level of LINC00152 was significantly higher in the 152 malignant lesions than in the paired paratumorous areas (*P* < 0.001, **Figure 1B**), which was consistent with the result of 2 independent cohorts from TCGA (www.cbioportal.org, n = 440, **Figures 2A, B**).

**Table 1:** Relationship between LINC00152 expression and clinicopathologic parameters of ovarian cancer patients.

Variables	Number (n = 152)	LINC00152 expression^a^	*P* value
Age (years)			0.125
<50	68	2.668 (0.186- 12.044)	
≥50	84	2.040 (0.181- 14.767)	
Tumor size			0.009*
<5 cm	53	1.701 (0.186- 7.071)	
≥5 cm	99	2.652 (0.181-14.767)	
Histology			0.451
High grade Serous	99	2.511 (0.186-14.767)	
Endometrioid	25	2.148 (0.181-12.044)	
Clear cell	21	2.024 (0.226-7.002)	
Mucinous	7	1.133 (0.330-2.562)	
Grades (Endometrioid)			0.219
I	14	1.326(0.181-3.896)	
II	6	3.321(0.410-7.200)	
III	5	3.043(0.370-12.044)	
FIGO Stage^b^			0.001*
I	37	1.249 (0.181-3.937)	
II	13	1.303 (0.362-2.724)	
III	97	2.743 (0.186-14.767)	
IV	5	4.710 (1.340-7.445)	
Vascular invasion			0.015*
Absent	89	1.973 (0.186-11.114)	
Present	38	3.210 (0.226-12.044)	
The great Omentum invasion			0.068
Absent	57	2.040 (0.181-11.114)	
Present	61	2.813 (0.407-12.044)	
Lymphatic metastasis			<0.001*
Absent	55	1.597 (0.181-5.450)	
Present	37	4.736 (1.104-14.767)	
Recurrence			0.010*
Absent	107	1.911 (0.181-9.579)	
Present	45	3.295 (0.410-14.767)	

^a^Median of relative expression with minimum–maximum percentile is recorded in parentheses.

^b^Tumor stage was obtained according to the FIGO (International Federation of Gynecology and Obstetrics) criteria.

*P < 0.05.

Next, we analyzed the correlation between LINC00152 expression and the clinicopathologic parameters of patients with EOC, both in our cohort and the cohorts from TCGA. First, the LINC00152 levels positively correlated with the advanced FIGO stage (*P* < 0.001, **Figure 1C**), consistent with the results of cohorts from TCGA (n = 440, **Figure 2C**). Second, the LINC00152 mRNA levels were increased in the patients with larger tumor sizes (>5 cm in diameter, *P* = 0.009, **Table 1**), increased vascular invasion (*P* = 0.015, **Table 1**) and with lymph node metastasis (*P* < 0.001, **Table 1**). Notably, the expression of LINC00152 detected by RT-qPCR did not correlate with the histological subtypes of EOC (high-grade serous, endometrioid, clear cell and mucinous types) (**Table 1**). Within the endometrioid subtypes, the LINC00152 mRNA levels did not correlate with the tumor grades (**Table 1**). When LINC00152 expression was divided into the high and low expression groups by the median value according to the previous studies [22,23], the distributions of patients with advanced FIGO stage (*P* < 0.001, **Figure 1C**), larger tumor size (*P* = 0.004, **Figure 2D**) and with lymph node metastasis (*P* < 0.001, **Figure 2E**) correlated with high expression of LINC00152.

We then elucidated the impacts of LINC00152 on patients’ clinical outcomes. The total median follow-up time for the patients who were still alive at the endpoint for analysis was 31.50 months. When dividing LINC00152 into the high and low expression groups by the median value, the median follow-up time for the patients who were still alive at the endpoint was 29.50 months in the high LINC00152 expression group and 32.00 months in the low expression group. The LINC00152 levels in recurrent cases were significantly increased (*P* = 0.010, **Table 1**), and high LINC00152 expression was associated with the recurrence (*P* < 0.001, **Figure 1D**). The Kaplan-Meier analysis showed that patients with high LINC00152 expression (n = 76) had significantly shorter disease-free survival (DFS) (*P* = 0.004; **Figure 1E**) and disease-specific (DSS) rates (*P* = 0.005; **Figure 1F**) compared with those with low expression (n = 76). These results corresponded with that of the cohorts from TCGA, which illustrated that high LINC00152 expression also correlated with poorer overall survivals (OS, *P* = 0.035, n = 440, **Figure 2F**). The univariate Cox analysis showed that lymph node metastasis, FIGO stage and LINC00152 expression all correlated with the DFS (*P* = 0.004, **Table 2**) and DSS (*P* = 0.002, **Table 3**). The multivariate analysis using the Cox proportional hazards model suggested that the LINC00152 level was an independent predictor for DFS (*P* = 0.029, **Table 2**) and DSS (*P* = 0.040, **Table 3**), along with FIGO stage (*P* = 0.026 for DFS, **Table 2**; *P* = 0.048 for DSS, **Table 3**). Furthermore, the receiver operating characteristics (ROC) curves indicated that both LINC00152 expression and FIGO stage showed similar AUC (area under curve) for DFS (*P* < 0.01, **Figure 1F**) and DSS (*P* < 0.01, **Figure 1F**), while the combination of these two independent predictors presented superior prognostic value (*P* < 0.01, **Figure 1F**). Taken together, these data suggest that abnormal LINC00152 expression predicts poor clinical outcomes for patients with EOC, and the combined analysis of LINC00152 expression with FIGO stage could serve as a more sensitive and specific prognostic marker for EOC.

**Table 2:** Univariate and multivariate analysis of clinicopathological factors for disease-free survival in ovarian cancer.

Variable	Univariate analysis		Multivariate analysis	
	HR (95% CI)	*P* ^a^	HR (95% CI)	*P* ^a^
Age (<50/≥50)	1.634 (0.905-2.949)	0.103		
Tumor size (<5 cm/≥5 cm)	1.999 (0.961-4.159)	0.064		
Vascular invasion (Present/Absent)	1.656 (0.859-3.195)	0.132		
The greater omentum invasion (Present/Absent)	1.304 (0.700-2.429)	0.403		
Lymph node metastasis				
(Present/Absent)	4.743 (1.991-11.299)	0.000*		
FIGO stage (III+IV/I+II)	4.169 (1.644-10.569)	0.003*	4.042 (1.183-13.812)	0.026*
LINC00152 (High/Low)	2.378 (1.277-4.426)	0.004*	5.196 (1.188-22.727)	0.029*

HR, Hazard ratio; 95% CI, confidence interval,

^a^All statistical tests were 2-sided. Significance level: *P < 0.05.

**Table 3:** Univariate and multivariate analysis of clinicopathological factors for disease-specific survival in ovarian cancer.

Variable	Univariate analysis		Multivariate analysis	
	HR (95% CI)	*P* ^a^	HR (95% CI)	*P* ^a^
Age (<50/≥50)	1.510 (0.742-3.073)	0.256		
Tumor size (<5 cm/≥5 cm)	2.156 (0.824-5.641)	0.117		
Vascular invasion (Present/Absent)	1.927 (0.889-4.178)	0.097		
The greater omentum invasion (Present/Absent)	1.030 (0.494-2.145)	0.938		
Lymph node metastasis				
(Present/Absent)	4.026 (1.691-9.588)	0.001*		
FIGO stage (III+IV/I+II)	1.884 (1.148-3.091)	0.005*	1.854 (1.120-3.069)	0.048*
*LINC00152* (High/Low)	3.108(1.448-6.671)	0.002*	4.544(1.034-19.969)	0.040*

HR, Hazard ratio; CI, confidence interval.

^a^All statistical tests were 2-sided. Significance level: *P < 0.05.

### LINC00152 Promotes Ovarian Tumor Cell Proliferation and Metastasis *In Vitro* and *In Vivo*

**Figure 3:**
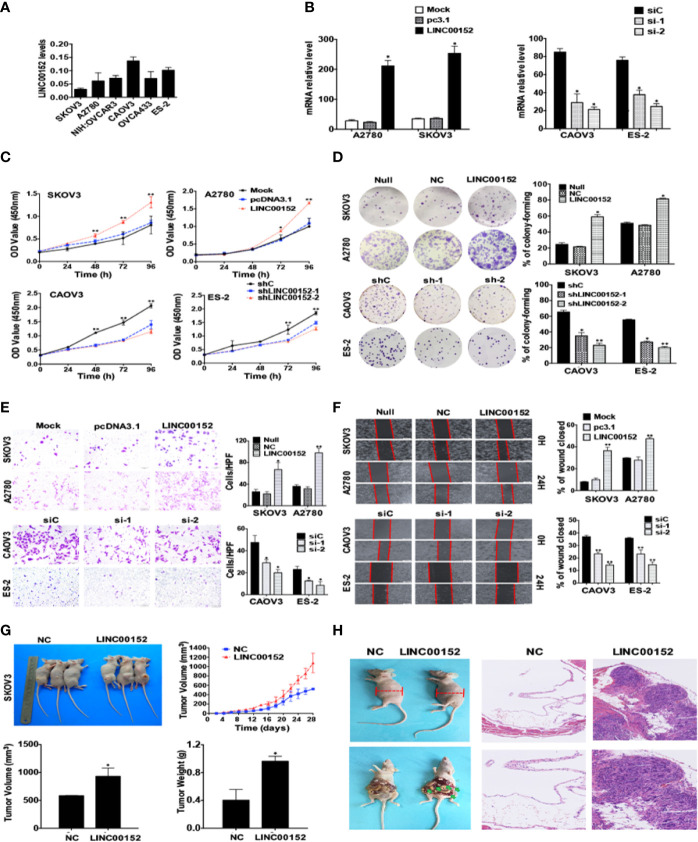
LINC00152 promotes ovarian cancer cell proliferation and metastasis *in vitro* and *in vivo.* **(A)** The endogenous mRNA levels of LINC00152 in ovarian cell lines collected in this study detected by RT-qPCR. **(B)** The mRNA levels of LINC00152 detected by RT-qPCR in A2780 and SKOV3 cells transfected with or without pcDNA3.1-LINC00152. *P < 0.01. (Left) The mRNA levels of LINC00152 detected by RT-qPCR in CAOV3 and ES-3 cells transfected with siC or siRNAs of LINC00152. *P < 0.01. (Right) **(C)** The CCK8 cell counting assays revealed the cell growth curves of NC and LINC00152-overexpressing SKOV3 and A2780 cells as well as shNC and LINC00152-knockdown ES-2 and CAOV3 cells at the indicated time points. Error bars are shown. *P < 0.01 and **P < 0.05. **(D)** Representative images of the colony-forming results of of NC and LINC00152-overexpressing SKOV3 and A2780 cells as well as shNC and LINC00152-knockdown ES-2 and CAOV3 cells for 14 days. *P < 0.01 and **P < 0.05. **(E)** Representative images (left) and quantification (right) of transwell invasion assays for the A2780 and SKOV3 cells overexpressed with LINC00152 as well as ES-2 and CAOV3 cells that were transfected with siLINC00152. Scale bars = 200 µm. *P < 0.01 and **P < 0.05. **(F)** Representative images (Left) and quantification (Left) of wound-healing assays for A2780 and SKOV3 cells overexpressed with LINC00152 as well as ES-2 and CAOV3 cells that were transfected with siLINC00152. Scale bars = 400 µm. *P < 0.05 and **P < 0.01. **(G)** Representative xenograft images (Top Left) and the statistical analytical graph (Top Right) of the xenograft tumor growth speed in nude mice injected with Lenti-NC and Lenti-LINC00152 overexpressing SKOV3 cells. The sizes (Bottom Left) and weights (Bottom Right) of xenografts were measured and analyzed. The error bars are shown. *P < 0.05. **(H)** The representative abdominal circumferences of nude mice injected with Lenti-NC and Lenti-LINC00152 overexpressing SKOV3 cells (Top Left). The metastatic lesions were shown by arrows (Bottom Left). The H&E images of metastatic lesions were also shown (Right). Top Right: Scale bars = 50 µm. Bottom Right: Scale bars = 25 µm.

To further study the biological effects of LINC00152 on EOC, we assessed LINC00152 mRNA levels in a panel of cell lines and chose the candidates A2780 and SKOV3 cells for overexpression while ES-2 and CAOV3 cells were selected for knockdown (**Figure 3A**). The efficiencies of overexpression and interference were confirmed by RT-qPCR (*P* < 0.01, **Figure 3B**). Then we investigated the potential effects of LINC00152 on EOC *in vitro* and *in vivo*. The CCK8 counting and colony-forming assays showed that overexpression of LINC00152 stimulated the proliferations of A2780 and SKOV3 cells (*P* < 0.05, **Figures 3C, D**), while knockdown of LINC00152 decelerated the growth of CAOV3 and ES-2 cells *in vitro* (*P* < 0.05, **Figures 3C, D**). Similarly, the transwell and wound-healing assays showed that overexpression of LINC00152 enhanced the mobilization and penetration of SKOV3 and A2780 cells while knockdown of LINC00152 reduced the invasion and migration of CAOV3 and ES-2 cells (**Figures 3E, F**). Finally, the *in vivo* xenograft models showed that overexpression of LINC00152 accelerated the growth and enlarged the sizes of xenografts (*P* < 0.05, **Figure 3G**); the metastasis models showed that overexpression of LINC00152 increased the abdominal circumferences of nude mice and caused more metastatic lesions in the abdominal organs (**Figure 3H**). These results suggest that LINC00152 promotes ovarian tumor growth and invasion both *in vitro* and *in vivo*.

### Identification of LINC00152 as the New Regulator of BCL6 Protein

**Figure 4:**
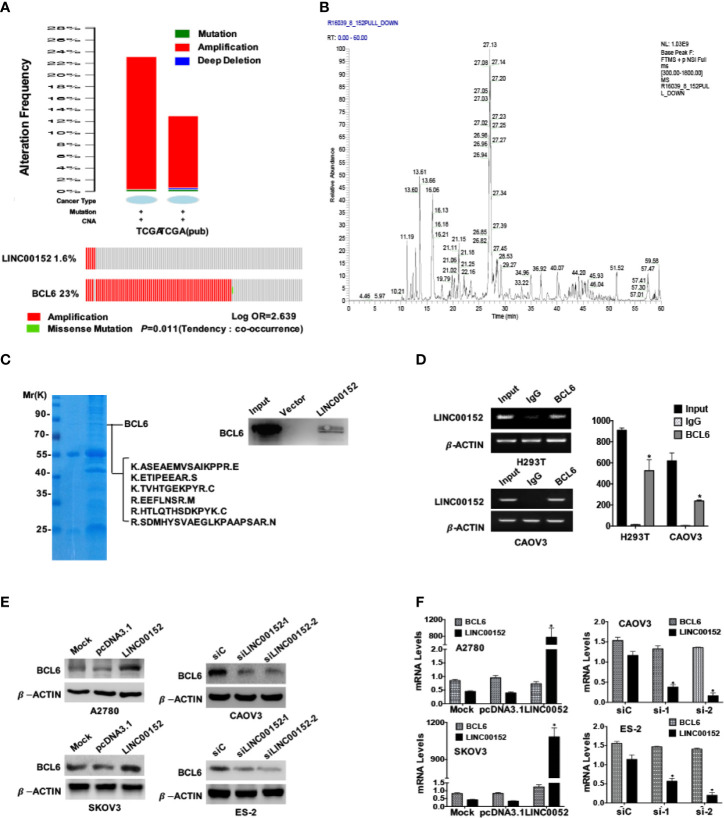
LINC00152 interacts with BCL6 protein. **(A)** Top: The status summary of LINC00152 and BCL6 in 2 independent cohorts from TCGA analyzed by www.cbioportal.org. The gene status included mutation, amplification and deep deletion. Bottom: The mutual exclusivity analysis of LINC00152 and BCL6 in 2 independent cohorts of ovarian cancer in TCGA. The Tendency was calculated by www.cbioportal.org. *P* < 0.05. **(B)** LINC00152-protein complex immunoprecipitated using the RNA-protein pulldown kit from the H293T cells. The protein bands of SDS-PAGE were cut and digested and subjected to MS LC-MS/MS analysis. The base peak spectra of all the binding targets of BCL6 were shown. **(C)** The Comassie blue stained SDS-PAGE presented the proteins pulled down by LINC00152 from CAOV3 cells, and the following L cmS/MS revealed the sequences from BCL6 in the pulldown complex (Left). The western blotting result by the independent RNA pulldown assays also showed that BCL6 was among the proteins binding to LINC00152 (Right). **(D)** The RNA immunoprecipitation assays showed the interaction between LINC00152 and BCL6 protein in H293T and CAOV3 cells. The cell lysates were used as the inputs and IgG as the negative control. The RT-qPCR products were both analyzed with RNA electrophoresis (Left) and quantified by statistical graphs (Right). *P < 0.01. **(E)** Western blot result showed the influences on BCL6 protein levels by either overexpression of LINC00152 in A2780 and SKOV3 cells or knockdown of LINC00152 in ES-2 and CAOV3 cells. **(F)** The RT-qPCR results showed no influences on BCL6 RNA levels either by overexpression of LINC0052 in A2780 and SKOV3 cell, or knockdown of LINC00152 in ES-2 and CAOV3 cells. *P < 0.01.

Next we analyzed the data of two cohorts in the TCGA and found that both LINC00152 and BCL6 genes were amplified in these cohorts (**Figure 4A**), and there was a significant co-expression tendency between these two genes (*P* = 0.011, **Figure 4A**). The LC-MS/MS spectrometry analysis identified the purified RNA-protein complex, which was pulled down by LINC00152 using the biotin RNA-protein pulldown assay in CAOV3 cells, contained the peptides of BCL6 (**Figures 4B, C** and **Table S1**). The spectrometry results were also confirmed by the western blotting result of RNA-protein pulldown products (**Figure 4C**). Besides, the RNA immunoprecipitation assay (RIP) using CAOV3 cells and H293T cells also showed that LINC00152 was bound by the endogenous BCL6 protein (**Figure 4D**). Moreover, overexpression of LINC00152 elevated the protein levels of BCL6 in A2780 and SKOV3 cells, while knockdown of LINC00152 reduced the protein levels of BCL6 in CAOV3 and ES-2 cells (**Figure 4E**). In contrast, the interference of LINC00152 failed to affect the mRNA levels of BCL6 accordingly (**Figure 4F**).

**Figure 5:**
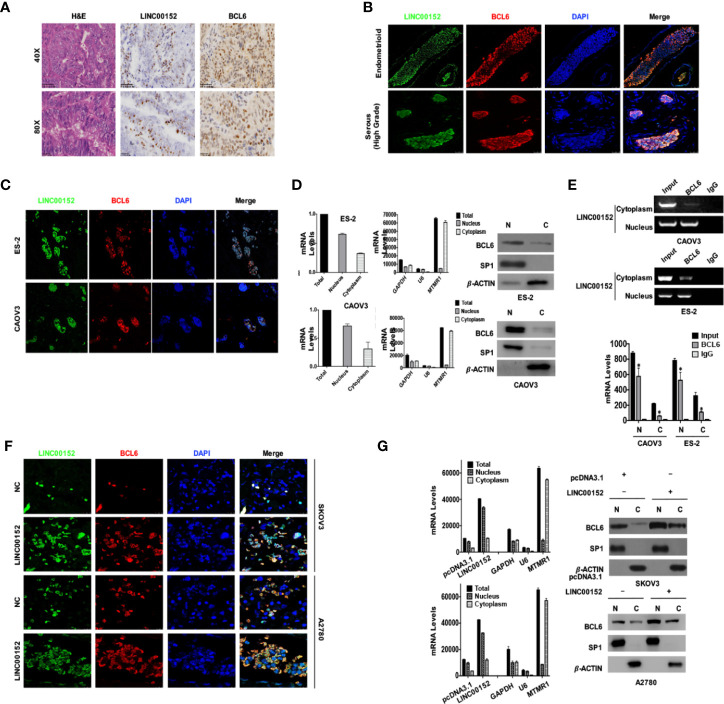
LINC00152 elevates the protein level of BCL6 both in nucleus and cytoplasm of ovarian tumor cells. **(A)** The RNA-protein dual staining results showed the staining of LINC00152 and BCL6 protein in ovarian cancers. Top: Scale bars = 50 µm. Bottom: Scale bars = 25 µm. **(B)** The dual staining of LINC00152 and BCL6 protein by RNAscope immunofluorescence in ovarian carcinomas of either emdometrioid (Top) or high-grade serous (Bottom) types. Scale bars = 25 µm. **(C)** The subcellular localizations of LINC00152 and BCL6 dual staining in ES-2 cells and CAOV3 cells detected by RNA-protein dual immunofluorescence. The LINC00152 mRNA probe (green) and anti-BCL6 antibody (red) were used. DAPI (blue) was used for the nucleus. **(D)** The nucleus-cytoplasm split RNA/protein extraction and detection of LINC00152 and BCL6 protein in ES-2 and CAOV3 cells. The RNA was detected by RT-qPCR and the protein was detected by western blot. GAPDH, U6 and MTMR1 were used as the controls of total RNA, nuclear RNA and cytoplasmic RNA. -ACTIN was used as the control for western blot. **(E)** The split nucleus-cytoplasm interaction of LINC00152 and BCL6 detected by RNA-protein immunoprecipitation. The cell lysates were used as the inputs and IgG as the negative control. The RT-qPCR products were both analyzed with RNA electrophoresis (Left) and quantified by statistical graphs (Right). *P < 0.01. **(F)** The dual staining of LINC00152 and BCL6 protein by RNAscope immunofluorescence in NC and LINC00152-overexpressing A2780 and SKOV3 cells. Scale bars = 10 µm. **(G)** The nucleus-cytoplasm split RT-qPCR results of LINC00152 and western blot results of BCL6 in NC and LINC00152-overexpressing A2780 and SKOV3 cells. The U6 was used as the cytoplasmic control and MTMR1 as the nuclear control for RNA nucleus-cytoplasm split RT-qPCR. SP1 was used as the nuclear control and -ACTIN as the cytoplasmic control for the nucleus-cytoplasm split western blot.

To further confirm the localization of LINC00152 and BCL6 protein, we performed RNA-Protein dual staining using the RNAscope technique (ACD, USA) in EOC tissues and tumor cell lines. The LINC00152 and BCL6 protein were expressed predominantly in the nucleus in the ovarian high-grade serous and endometrioid carcinoma tissues (**Figure 5A**); however, the sensitive immunofluorescence showed that there were still residual amounts of LINC00152 and BCL6 proteins localized in the cytoplasm (**Figure 5B**). The following immunofluorescence and nuclear-cytoplasmic RT-qPCR/western blotting assays in both CAOV3 and ES-2 cells also proved that LINC00152 and BCL6 protein were co-expressed mainly in the nucleus but were also expressed at low levels in the cytoplasm (**Figures 5C, D**). More importantly, the split nuclear/cytoplasmic RIP assay showed that LINC00152 could interact with BCL6 protein both in the nucleus and cytoplasm of CAOV3 and ES-2 cells (**Figure 5E**). In addition, overexpression of LINC00152 in A2780 and SKOV3 cells enhanced the expressions of BCL6 protein both in the nucleus and cytoplasm, as observed by the immunofluorescence and RT-qPCR/western blotting assays (**Figures 5F, G**). These findings suggest that LINC00152 interacts with the co-localized BCL6 protein and elevates its level in EOC.

### LINC00152 Stabilizes BCL6 Protein From Ubiquitination by Binding the Specific Sites

**Figure 6:**
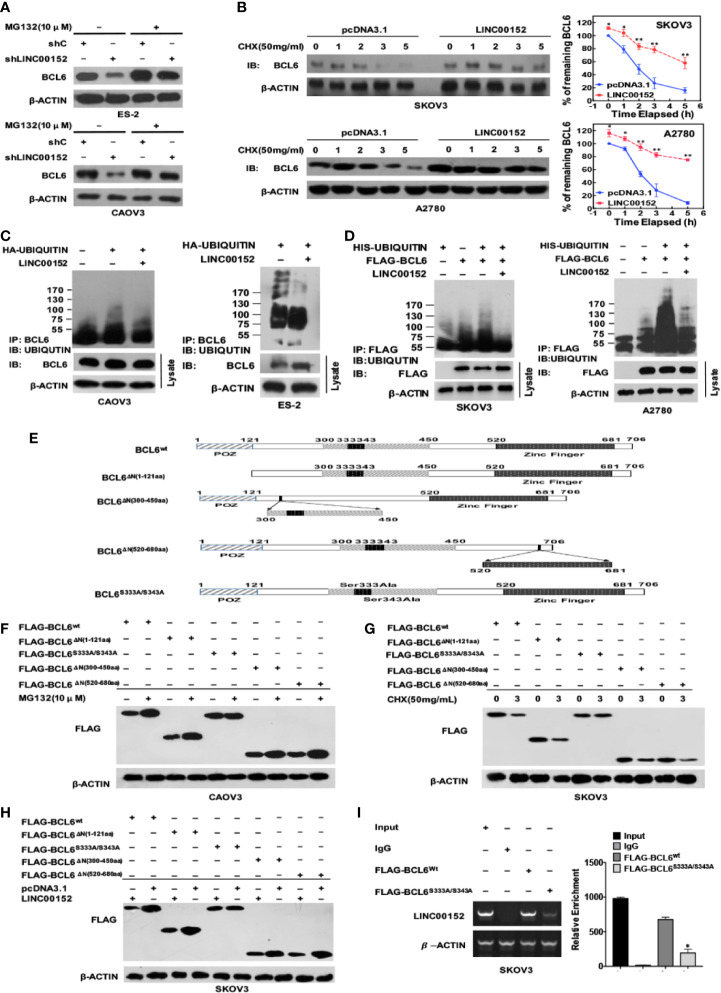
LINC00152 elevates the level of BCL6 protein by binding its specific sites and suppressing its ubiquitination. **(A)** The western blot results showed the rescued protein levels of BCL6 by MG132 (10 µM) in NC and LINC00152-knockdown ES-2 and CAOV3 cells. **(B)** The western blot results showed the prolonged half-life of BCL6 protein in NC and LINC00152-overexpressing A2780 and SKOV3 cells under the influence of CHX (50 mg/mL) at indicated time points. (Left) The analytic graphs of percentage of BCL6 at the indicated time points in SKOV3 and A2780 cells transfected with pcDNA3.1 and LINC00152 under the influence of CHX. The Protein level of BCL6 was quantified by ImageJ. *P < 0.05 and **P < 0.01. (Right). **(C)** The *in vivo* ubiquitination assays showed the endogenous polyubiquinated-BCL6 protein in NC and LINC00152-overexpressing CAOV3 and ES-2 cells. **(D)** The *in vivo* ubiquitination assays showed the exogenous polyubiquinated-BCL6 protein in NC and LINC00152-overexpressing SKOV3 and A2780 cells. **(E)** The schematic diagram of full-length BCL6 protein and its mutants used in the study. The truncated domains and the mutated sites were shown. **(F)** The western blot results showed the protein levels of wild-type BCL6 and its mutants under the influence of MG 132 (10 µM) in CAOV3 cells. **(G)** The western blot results showed the protein levels of wild-type BCL6 and its mutants at the indicated time points under the influence of CHX (50 mg/mL) in CAOV3 cells. **(H)** The western blot results showed the protein levels of wild-type BCL6 and its mutants under the influence of LINC00152 overexpression in SKOV3 cells. **(I)** The RNA immunoprecipitation assays showed the levels of LINC00152 precipitated by Flag-tagged wild-BCL6 and its mutant Flag-tagged BCL6^S333A/S343A^. The RT-qPCR products were turned to either electrophoresis or quantification by graph. *P < 0.01.

Based on the results that LINC00152 altered BCL6 on the protein level but not at the mRNA level (**Figures 4E, F**), there existed the possibility that LINC00152 stabilized BCL6 post-translationally. We used the protein inhibitor MG132 to successfully rescue the reduced levels of BCL6 protein caused by the knockdown of LINC00152 in CAOV3 and ES-2 cells (**Figure 6A**). Meanwhile, we observed that overexpression of LINC00152 prolonged the semi-life of BCL6 protein under the influence of the eukaryotic protein synthesis inhibitor Cycloheximide (CHX) in A2780 and SKOV3 cells (**Figure 6B**, *P* < 0.05). Furthermore, the ubiquitination assay confirmed that the overexpression of LINC00152 could reduce the levels of endogenous poly-ubiquitinated BCL6 in CAOV3 and ES-2 cells (**Figure 6C**) as well as the exogenous poly-ubiquitinated BCL6 in SKOV3 and A2780 cells (**Figure 6D**). Taken together, these data suggest that LINC00152 elevates the protein level of BCL6 by inhibiting its ubiquitination.

Niu et al. first observed that among the region N310aa-N450aa of BCL6 protein, there are several potential sequences related to the ubiquitin/proteosome pathway (29), and Kurosu et al. found that Ser333/S343 of BCL6 is involved in the proteasome-dependent degradation (30). Besides, the BTB domain (N-terminal 32-99aa) might form dimers to induce the degradation of BCL6 (31). Based on these findings, we constructed the corresponding plasmids of mutated and truncated BCL6 (**Figure 6E**). We observed that MG132 inhibited the degradation of wild-type BCL6 and the truncated BCL6 proteins including BCL^ΔN(1-121aa)^, BCL^ΔN(520-680aa)^ and BCL ^ΔN(300-450aa)^ but not the mutant BCL6^S333A/S343A^ (**Figure 6F**). In contrast, under the influence of CHX, the wild-type BCL6 and all the truncated proteins of BCL6 (BCL^ΔN(1-121aa)^, BCL^ΔN(520-680aa)^) and BCL ^ΔN(300-450aa)^ were degraded but not the mutant BCL6^S333A/S343A^ (**Figure 6G**). When LINC00152 was overexpressed, only the mutant BCL6^S333A/S343A^ failed to show the elevation of protein level (**Figure 6H**). Finally, the RIP assay showed that the binding between LINC00152 and mutant BCL6^S333A/S343A^ was significantly reduced compared with the binding of LINC00152 to wild-type BCL6 (*P* < 0.01, **Figure 6I**). These data suggest that LINC00152 binds to Ser333/Ser343 of BCL6 protein and elevates its protein level by suppressing its ubiquitination.

### LINC00152 Promotes Ovarian Tumor Proliferation and Invasion in a BCL6-Mediated Manner

**Figure 7:**
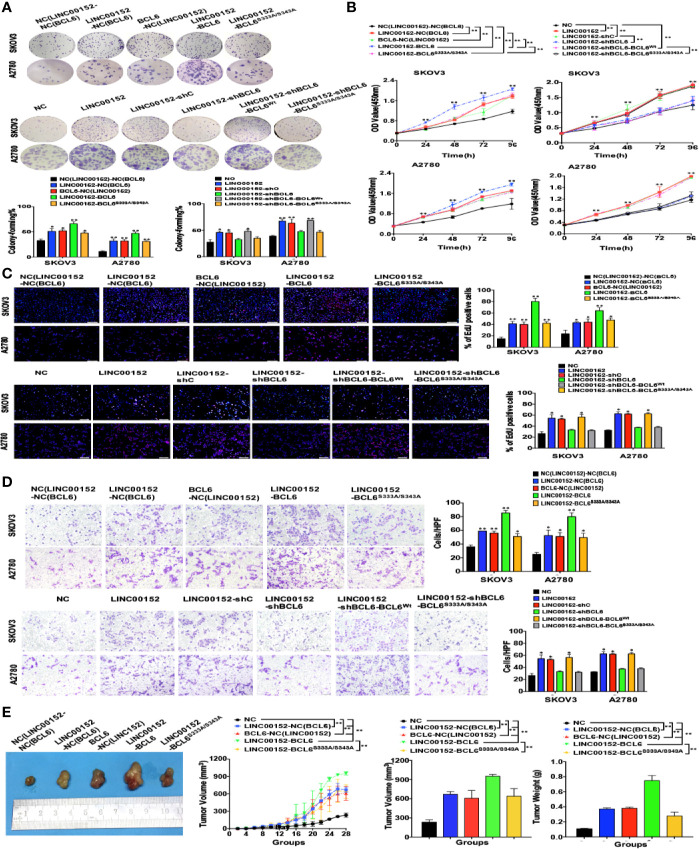
LINC00152 promotes ovarian cancer progression in a BCL6-mediated manner. **(A)** The western blot results of BCL6, SNAIL, N-CADHERIN and CCNB1 in the NC, LINC00152-overexpressing, LINC00152-shNC and LINC00152-shBCL6 SKOV3, and A2780 cells. **(B)** Left: The representative images of colony-forming (Left) of NC, LINC00152-overexpressing, LINC00152-shNC and LINC00152-shBCL6 SKOV3, and A2780 cells for 14 days. *P < 0.05 and **P < 0.01. Right: The CCK8 counting curves of NC, LINC00152-overexpressing, LINC00152-shNC and LINC00152-shBCL6 SKOV3, and A2780 cells with the indicated time points. *P < 0.05 and **P < 0.01. **(C)** The representative images of EdU immunofluorescence (Top) and transwell assay (Bottom) in NC, LINC00152-overexpressing, LINC00152-shNC and LINC00152-shBCL6 infected SKOV3 and A2780 cells. The percentage of EdU positive cells were graphed under 100× and calculated under 200× magnification. The penetrated cells were graphed under 200× and counted under 400× magnification. *P < 0.05 and **P < 0.01. **(D)** The xenograft tumorigenesis results of NC, LINC00152-overexpressing, LINC00152-shNC and LINC00152-shBCL6 infected SKOV3 cells. Tumors were photographed and the speed of tumor growth was illustrated as curves, and the weights of tumors were calculated and analyzed. **P < 0.01.

We then investigated whether LINC00152 exhibited its functions in EOC in a BCL6-mediated manner. First, we found that knockdown of BCL6 in the LINC00152-overexpressing A2780 and SKOV3 cells led to the reduction of metastasis-related genes such as SNAIL and N-CADHERIN, as well as the proliferation-related gene CCNB1 (**Figure 7A**). The CCK-8, colony-forming and EdU assays showed that overexpression of LINC00152 stimulated the proliferation of SKOV3 and A2780 cells, which was impaired by simultaneous knockdown of BCL6 (*P* < 0.05, **Figure 7B**). The transwell assay also demonstrated that knockdown of BCL6 partially attenuated the effects of overexpression of LINC00152 on ovarian tumor cell invasion (*P* < 0.05, **Figure 7C**). Consistently, the *in vivo* xenograft models confirmed that knockdown of BCL6 partially abolished the accelerated tumor growth and the increased tumor size/weight induced by overexpression of LINC00152 (*P* < 0.05, **Figure 7D**). All these data suggest that LINC00152 prompts ovarian tumor progression in a BCL6-mediated manner.

### Ser333/Ser343 of BCL6 Is Essential to the Oncogenic Functions of LINC00152-BCL6 Interaction

**Figure 8:**
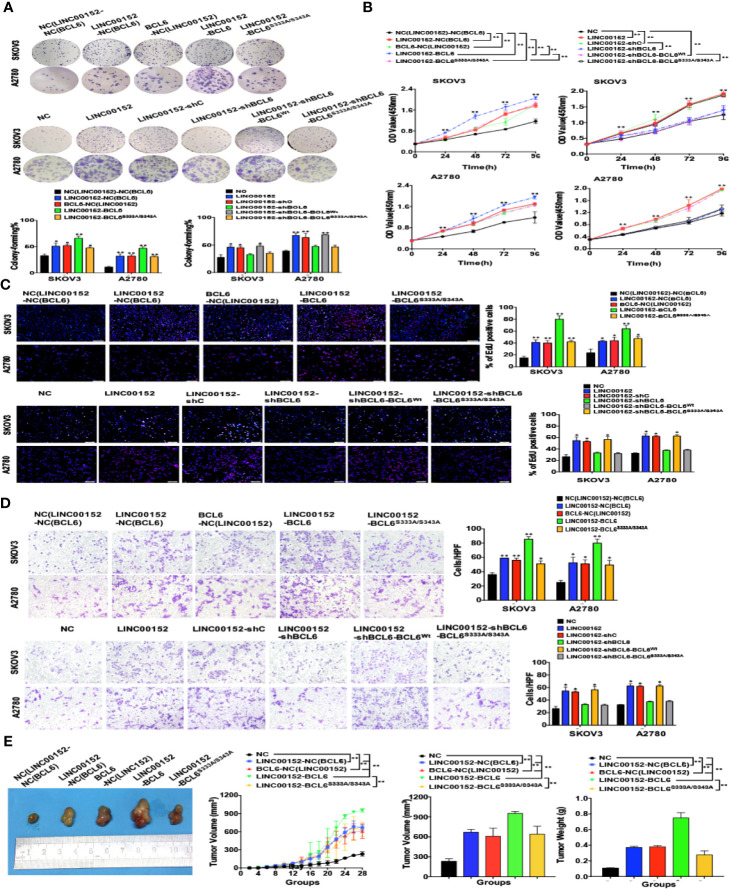
Ser333/Ser343 is essential to LINC00152-BCL6 facilitated ovarian tumor progression. **(A)** The representative images of colony-forming SKOV3 and A2780 cells infected with the lentivirus plasmids(Top: NC, LINC00152-NC(BCL6), BCL6-NC(LINC00152), LINC00152-BCL6, and LINC00152-BCL6^S333A/S343A^; Bottom: NC, LINC00152, LINC00152-shC, LINC00152-shBCL6, LINC00152-shBCL6-BCL^Wt,^ and LINC00152-shBCL6-BCL6^S333A/S343A^) for 14 days. *P < 0.05 and **P < 0.01. **(B)** The CCK8 counting curves of SKOV3 and A2780 cells infected with the lentivirus plasmids (Left: NC, LINC00152-NC(BCL6), BCL6-NC(LINC00152), LINC00152-BCL6 and LINC00152-BCL6^S333A/S343A^; Right: NC, LINC00152, LINC00152-shC, LINC00152-shBCL6, LINC00152-shBCL6-BCL^Wt,^ LINC00152-shBCL6-BCL6^S333A/S343A^) with the indicated time points. **P < 0.01. **(C)** The representative images of EdU immunofluorescence in SKOV3 and A2780 cells infected with the lentivirus plasmids(Top: NC, LINC00152-NC(BCL6), BCL6-NC(LINC00152), LINC00152-BCL6 and LINC00152-BCL6^S333A/S343A^; Bottom: NC, LINC00152, LINC00152-shC, LINC00152-shBCL6, LINC00152-shBCL6-BCL^Wt,^ and LINC00152-shBCL6-BCL6^S333A/S343A^). The percentage of EdU positive cells were graphed under 100× and calculated under 200× magnification. *P < 0.05 and **P < 0.01. **(D)** The representative images of transwell assay in SKOV3 and A2780 cells infected with the lentivirus plasmids(Top: NC, LINC00152-NC(BCL6), BCL6-NC(LINC00152), LINC00152-BCL6 and LINC00152-BCL6^S333A/S343A^; Bottom: NC, LINC00152, LINC00152-shC, LINC00152-shBCL6, LINC00152-shBCL6-BCL^Wt^, and LINC00152-shBCL6-BCL6^S333A/S343A^). The penetrated cells were graphed under 200× and counted under 400× magnification. *P < 0.05 and **P < 0.01. **(E)** The xenograft tumorigenesis results of NC, LINC00152-NC(BCL6), BCL6-NC(LINC00152), LINC00152-BCL6, and LINC00152-BCL6^S333A/S343A^ infected SKOV3 cells. Tumors were photographed and the speed of tumor growth was illustrated as curves, and the weights of tumors were calculated and analyzed. **P < 0.01.

Finally, we identified the indispensability of the specific sites of BCL6 to the biological functions of LINC00152-BCL6 interaction. The CCK8, colony-forming, EdU and transwell assays revealed that the enhanced proliferation and invasion of SKOV3 and A2780 cells induced by overexpression of LINC00152 could be further boosted by co-overexpression of the wild-type BCL6 but not the mutant BCL6^Ser333/Ser343^; meanwhile, overexpression of exogenous BCL6 ^Ser333/Ser343^ failed to rescue the abolished proliferation and invasion caused by knockdown of endogenous BCL6 in LINC00152-overexpressing tumor cells (*P* < 0.05, **Figures 8A**–**D**). Finally, the xenograft models indicated that the accelerated growth speed and increased size/weight of xenografts induced by overexpression of LINC00152 could be amplified by co-overexpression of the wild-type BCL6 but not the mutant BCL6^Ser333/Ser343^ (*P* < 0.05, **Figure 8E**). Taken together, these results suggest that the interaction between LINC00152 and the Ser333/Ser343 residues of BCL6 is essential to the oncogenic functional fulfilment of LINC00152 in EOC.

## Discussion

In this study, we found that the mRNA level of LINC00152 was abnormally increased in the ovarian tumor tissues and cell lines, and was accompanied with dysregulated biological functions, poorer clinical outcomes and increased cell proliferation and metastasis. Moreover, we provided the first-hand evidence of the post-translational modification on the degradation of BCL6 by LINC00152, which directly promoted the progression of EOC. Thus, our study details the downstream targets of LINC00152 and the molecular mechanisms of BCL6-related lncRNAs in the tumorigenesis of EOC.

Located at 2p11.2, LINC00152 has been reported as an oncogenic lncRNA in multiple somatic malignancies (32, 33). We formerly reported that LINC00152 predicted negative prognosis and prompted tumor invasion in renal clear cell carcinoma and lung adenocarcinoma (11, 12). In this study, we described the dysregulated increase of LINC00152 expression in EOC tissues as well as the correlation between its abnormal expression and poor prognosis. In addition, high LINC00152 expression was independently predicted the risks of poorer DFS and DSS, and the combination of LINC00152 and FIGO stage in ROC curve analysis could offer better predictive value of patients’ survivals compared with FIGO staging or LINC00152 alone. Although the FIGO stage has been considered as the crucial risk factor for the prognosis of EOC (34), the survival rates of patients at the same stage are different. Our data suggested that introduction of LINC00152 expression into the stratified analysis might offer more precise prediction of risks of recurrence and mortality, and might also partially explain the differences in survival of patients at same FIGO stages. Based on the occult invasiveness of EOC, it would be of clinical value to apply LINC00152 as a regular monitoring factor postoperatively for the patients. Future studies need to focus on the correlation of LINC00152 expression in serum with that in tissues, which might simplify the methods of detection and monitoring in patients.

Previous studies have found that LINC00152 competes as a ceRNA with miRs in hepatic and cervical cancers (32, 35), and is related to the regulation of signaling pathways such as Wnt/β-Catenin pathway (35). In contrast, our study revealed a post-translational mechanism of LINC00152 by stabilizing BCL6 from ubiquitination. Moreover, depletion of BCL6 abolished the promotion of ovarian tumor progression caused by LINC00152, suggesting that the oncogenic activities of LINC00152 is a reflection of the accumulation of BCL6 protein. Niu et al. found that the degradation-targeted sequences are located in the same region as the MAPK putative phosphorylation sites are embedded, among which Serine333 and Serine343 are listed. However (36), Kurosu et al. reported that the MEK inhibitor failed to rescue the rapid degradation of BCL6 in large B lymphoma (30), and the BTB domain (N-terminal 32-99aa) might form dimers to induce the FBXL17-conducted degradation (31), suggesting that the ubiquitination of BCL6 might refer to other mechanisms apart from phosphorylation. Intriguingly, our study showed that LINC00152 nearly lost the ability to bind to the mutant BCL6 (S333A/S343A) and overexpression of LINC00152 failed to increase the protein level of BCL6^S333A/S343A^. Therefore, the mechanisms for LINC00152 to inhibit the ubiquitination of BCL6 might be as follows: (1) LINC00152 occupies Ser333/Ser343 of BCL6 to inhibit the ERK-induced phosphorylation as well as the proteosome-dependent ubiquitination; (2) the interaction of LINC01152 and Ser333/Ser343 of BCL6 disrupts the nearby domains for the conjugation of ubiquitin and ubiquitination-related kinases, thus stabilizing BCL6 protein from the ubiquitin/proteosome degradation. Moreover, LINC00152-BCL6 interaction might recruit additional miRNAs to cooperatively fulfil the functions. Future studies need to focus on these aspects to map the network centered by LINC00152-BCL6 interaction.

In summary, we found that LINC00152 is an applicable independent prognostic predictor of EOC, and its abnormal expression facilitates ovarian tumor proliferation and invasion by binding to Ser333/Ser343 of BCL6 to stabilizing its protein from ubiquitination. Our data might be crucial to the exploration of molecular mechanisms of lncRNA-protein interactions as well as to the identification of novel pharmacological candidates for ovarian cancer therapy.

## Data Availability Statement

Publicly available datasets were analyzed in this study. This data can be found here: www.cbioportal.org.

## Ethics Statement

The studies involving human participants were reviewed and approved by The Ethics Committee of Obstetrics and Gynecology Hospital of Fudan University. The patients/participants provided their written informed consent to participate in this study. The animal study was reviewed and approved by Shanghai Medical Experimental Animal Care Commission.

## Author Contributions

YW was the sponsor and designed the project. TC, MX, PW, JL, and LL were responsible for the part of experiments. SW and WW organized the data and composed the paper. All authors contributed to the article and approved the submitted version.

## Funding

This study was supported by National Natural Science Foundation of China (no. 81602269).

## Conflict of Interest

The authors declare that the research was conducted in the absence of any commercial or financial relationships that could be construed as a potential conflict of interest.
