# Supplementary material for: LINC00152 Promotes Tumor Progression and Predicts Poor Prognosis by Stabilizing BCL6 From Degradation in the Epithelial Ovarian Cancer
Source: Front Oncol. 2020 Nov 12;10:555132. doi: 10.3389/fonc.2020.555132 (PMC7690314; doi:10.3389/fonc.2020.555132)
Supplement: Supplementary file 1 [file Table_1.docx]

Supplementary Material

1. **Supplementary Methods**

**1.1 Cell culture, plasmids and reagents**

Human ovarian carcinoma cell lines H293T, A2780, CAOV3, ES-2, HEY, OVCA433, SKOV3 and NIH: OVCAR3 were purchased from IBS Cell bank of Fudan University and Cell bank of Shanghai Institute, Shanghai, China. All cells were grown and maintained in either DMEM or RPMI-1640 medium (Lot. 12633-012, GIBCO, U.S.A.) supplemented with 10% fetal bovine serum (FBS, Lot.10099-141-FBS, GIBCO, USA) and 1% penicillin and streptomycin, maintain at 37°C in a humidified atmosphere with 5% CO_2_. Stable A2780 and SKOV3 cells infected with Lenti-LINC00152-IRES-EGFP constructs were additionally grown in 4 μg/mL or 2 μg/mL Blasticidin S (Lot. A11139-02, Life, USA). The stable Lenti-shLINC00152 CAOV3 and ES-2 cell line transfected with Lenti-shLINC00152-GV112-GFP-Puro, Lenti-BCL6 SKOV3 and A2780 cells with Lenti-BCL6-GV112-GFP-Puro, and Lenti-BCL6^S333A/S343A^ SKOV3 and A2780 cells with Lenti - BCL6^S333A/S343A^ -GV112-GFP-Puro were maintained with 0.5 µg/mL puromycin (Lot. A1113803, Life, USA). The information for specific plasmids and reagents are listed in the **Table S4**.

**1.2 *In* *vitro* proliferation assays**

Cell proliferation was indirectly assayed using the CCK-8 kit (Dojindo, Japan) and cell-colony forming assays. The CCK-8 kit stains living cells. Approximately 5×10^3^ cells in 100μl were incubated in triplicate in 96-well plates. At 0h, 24h, 48h, 72h, and 96h, the CCK-8 reagent (10 L) was added to each well and incubated at 37°C for 3h. The optical density at 450nm was measured using an automatic microplate reader (Synergy4; BioTek, Winooski, VT, USA). As to the colony forming assay, the cells were plated in 6-well plates (800 cells per plate) and cultured for 10 days. Colonies were stained with 0.1% crystal violet for 30 min after fixation with ethanol for 30 min.

EdU imaging system was used to visualize and measure cell proliferation by counting the percentage of cells in progress of replication of DNA. 72h after transfection, cells were incubated with 10 μM EdU solution for 2 h and fixed with 3.7% Formaldehyde (Sigma) and penetrated with 0.5% triton X-100 (Sigma) for 20 min. Cells were stained with EdU/Alexa Fluor Azide 594 for 30 min followed by Hoechst 33342(1:2000) for another 30 min and imaged at 100× and counted at 200× under the fluorescence microscopy (IX51, Olympus, Japan).

**1.3 *In* *vitro* migration and metastasis assay**

Cell-wounding assays were used to evaluate the cell mobility. The cells were grown in 6-well plates until confluence. A cell scratch-wound was generated by scraping with a 10 µL tip. After 12h, the wounded cells were photographed, and cell migration was assessed by measuring gap sizes in multiple fields.

Transwell assay was used to assess cell invasion (Corning Co. Ltd., USA). The lower chambers were pre-coated with 100 μL Matrix gel (#354234, BD Bioscience, USA) for 30 min. 24h after transfection, cells were seeded on the upper chamber at 3.0×10^4^/well in serum-free medium. Medium containing 20% fetal bovine serum medium was applied to the lower chamber as chemo-attractant. After 24h incubation at 37°C, cells which invaded through the matrix gel and adhered to the lower surface of the filter were fixed with ethanol, stained with 0.5% crystal violet, photographed at 200×, and counted at 400× in 10 different fields to determine the average number of cells (BX51, Olympus, Japan).

**1.4 Immunochemistry**

Paraffin sections of the 200 cases with ovarian carcinomas were prepared and incubated overnight at 37°C with primary antibodies against BCL6 (Cell Signaling, USA) and LINC00152. Standard avidin-biotin immunochemical analysis of the sections was performed. The staining results were evaluated by at least three certified pathologists and the staining in either the nucleus or the nucleus-cytoplasm was considered as positive for BCL6.

**1.5 Ubiquitination assays**

For the *in vivo* ubiquitination/deubiquitination assays, cells were treated with MG132 (10 μM) for 3 hours and lysed. The lysates were immunoprecipitated by anti-Flag or anti-BCL6 and immunoblotted by anti-ubiquitin. The protocol of the *in vitro* deubiquitination assays was in accordance with our previous study.

**1.6 RNA isolation and quantitative RT-PCR**

Total RNA was extracted from tissue samples and cells using TRIzol (Invitrogen, Carlsbad, CA, USA) according to the manufacturer's protocol. The reverse transcription reactions were conducted using a PrimeScript^®^ RT reagent Kit (Takara, Dalian, China); the quantitative PCR reactions were then performed using SYBR^®^ Premix Ex TaqTM (Takara, Dalian, China), as previously described^[44]^. Glyceraldehyde-3-phosphate dehydrogenase (GAPDH) was included as the endogenous control to normalise the data. The primer sequences are listed in Supplementary **Table S5**.

**1.7 RNA fractionation and isolation**

The nuclear and cytoplasmic RNAs were extracted and isolated with Protein and RNA isolation systems (PARIS, Ambion, Thermo Fisher, USA) to determine the localizations of BCL6 protein and LINC00152 mRNA. The assays were performed according to the manufacturer’s instructions. Western blotting and RT-qPCR were used to quantify the expressions of BCL6 protein and LINC00152 mRNA.

**1.8 RNA Binding ProteinImmunoprecipitation (RIP) assays**

The RIP assays were conducted as previously described.

1. **Supplementary Tables**

**Supplementary Table 1.** Mass spectrometry of the proteins pulled down by LINC00152 in CAOV3 cells

| Number | Protein name | CoverPercent  Diff (MH+) |
| --- | --- | --- |
| 1 | BCL6 | 39.83% |
| 2 | RCC2 | 24.88% |
| 3 | EEF2 | 23.08% |
| 4 | HSPA1L | 20.99% |
| 5 | DSG1 | 24.34% |
| 6 | HRNR | 14.39% |
| 7 | DHX9 | 7.09% |
| 8 | FASN | 5.73% |
| 9 | EIF4G1 | 2.04% |

**Supplementary Table 2.** Reagents and antibodies

|  | **Cal.** | **Manufacturer** |
| --- | --- | --- |
| BCL6 | #14895 | Cell Signaling |
| UBIQUITIN | #3933S | Cell Signaling |
| FLAG | M2 monoclonal | Sigma Alderich |
| β-ACTIN | #4970 | Cell Signaling |
| MG132 | Lot. C2211 | Sigma Alderich |
| CHX | Lot. C7698 | Sigma Alderich |
| RNAScope 2.5HD | #322310 | Advanced Cell Diagnostics |
| RIPA lysis buffer | Lot. 89901 | Thermo Scientific |
| Puromycin | P7130-1G | Sigma Alderich |
| Lipofectamine 2000 | Lot. 11668-027 | Life Technology |

**Supplementary Table 3.** Primers used in the study

| **Gene** | **Primer Sequences and Targeting Sequences for siRNA or shRNA** |
| --- | --- |
| BCL6 | 5’-AACAAGCTTATGAAAACTAGCCCCCGTCGG-3’ (forward) |
|  | 5’-AAAGGATCCCTACTGTAGCTCAGGAATAAAC-3’ (reverse) |
| LINC00152 | 5’- TGAGAATGAAGGCTGAGGTGT -3’(forward) |
|  | 5’- GCAGCGACCATCCAGTCATT-3’(reverse) |
| shLINC00152-1 | GG TCTGGTCGGTTTCCCATTTdTdT |
| (siLINC00152-2) |  |
| shLINC00152-2 | GGAGAUGAAACAGGAAGCUdTdT |
| (siLINC00152-3) |  |
| Scramble | UUCUCCGAACGUGUCACGUdTdT |
